# Cyberbullying during COVID-19 lockdowns: prevalence, predictors, and outcomes for youth

**DOI:** 10.1007/s12144-023-04394-7

**Published:** 2023-02-18

**Authors:** Raquel António, Rita Guerra, Carla Moleiro

**Affiliations:** CIS-IUL, ISCTE- Instituto Universitário de Lisboa, Avª das Forças Armadas, Ed. ISCTE, CIS-IUL, Lisboa, 1649-026 Portugal

**Keywords:** Cyberbullying, COVID-19, Lockdowns, Social support, Suicidal ideation

## Abstract

The COVID-19 pandemic posed additional challenges to the safety and well-being of young people who were forced to engage in online learning, spending more time than ever online, and cyberbullying emerged as a notable concern for parents, educators, and students. Two studies conducted online examined the prevalence, predictors, and outcomes of cyberbullying episodes during the lockdowns due to the outbreak of COVID-19 in Portugal. Study 1 (*N* = 485) examined the prevalence of cyberbullying among youth during the first lockdown period in 2020, focusing on predictors, symptoms of psychological distress and possible buffers of the effects of cyberbullying. Study 2 (*N* = 952) examined the prevalence of cyberbullying, predictors, and symptoms of psychological distress during the second lockdown period in 2021. Results revealed that most participants experienced cyberbullying, symptoms of psychological distress (e.g., sadness and loneliness) during the lockdowns were higher for those who experienced than for those who did not experience cyberbullying, and those who experienced cyberbullying with higher levels of parental and social support showed lower levels of symptoms of psychological distress (i.e., suicidal ideation). These findings contribute to the existing knowledge on online bullying among youth, specifically during COVID-19 lockdowns.

**Supplementary information:**

The online version contains supplementary material available at 10.1007/s12144-023-04394-7.

The use of information and communication technologies (ICT) is associated with several psychological and social benefits (e.g., increased personal well-being, and increased students’ opportunities for social interaction and collaborative learning experiences; Bastiaensens et al., 2015; Li, 2007). However, the extensive use of communication technologies has also brought risks, such as the potential for aggressive behavior that is commonly labelled as cyberbullying (e.g., Kowalski et al., 2014). Cyberbullying is a specific type of bullying that involves using technology and digital media to harass, threaten or victimize, intentionally, repeatedly and over time another individual (e.g., DeSmet et al., 2016; Smith et al., 2008). This type of bullying, different than traditional bullying, can happen anywhere and anytime, beyond school gates, by known and unknown people, and makes possible the anonymity of the aggressor. Similar to traditional bullying, it also has several negative effects on the victims, such as anxiety symptoms (e.g., Bastiaensens et al., 2015; Elipe et al., 2018; Schneider et al., 2012).

During the lockdowns stemming from the COVID-19 pandemic, experts warned that millions of children and young people were vulnerable to experiencing cyberbullying, as schools were closed and replaced by online learning platforms (e.g., Armitage, 2021; Fore, 2020). To date, some studies have investigated the prevalence of cyberbullying related to the COVID-19 pandemic but focusing mainly on adults, and on East and Southeast Asian individuals as they were more likely to be the target of discrimination and hate speech due to COVID-19 (e.g., Alsawalqa, 2021; Barlett et al., 2021; Yang et al., 2022). Thus, the prevalence and symptoms of psychological distress of adolescents and young adults who experienced cyberbullying during online learning periods are still unclear. Extending previous research focusing on adults, in the current research we examine the prevalence of cyberbullying among Portuguese students, its predictors and outcomes (i.e., symptoms of psychological distress), as well as potential buffering factors (e.g., social support). Specifically, the following research questions were examined: (a) is the cyberbullying experience during the COVID-19 lockdowns related to symptoms of psychological distress in adolescents and young adults?; (b) is there an association between sex, educational level, sexual orientation, socioeconomic status, and cyberbullying involvement?; and (c) do social and parental support buffer the negative relation between cyberbullying victimization and youth well-being (i.e., psychological distress)?.

## Cyberbullying and the COVID-19 pandemic

Cyberbullying has been associated with the increased use of electronic devices (e.g., computers and mobile phones; Smith et al., 2006) and occurs through different media channels (text message, email, social network sites, chat rooms and online games; Bastiaensens et al., 2015; Kowalski et al., 2014; Smith et al., 2006). Like traditional bullying, can take different forms (e.g., harassment, exclusion, outing, trickery, cyber-stalking, sexting) and has also been related to several negative psychosocial, physical, and mental health consequences, such as depression, suicidal attempts, anxiety, loneliness, substance abuse, and lower academic achievement (e.g., Bastiaensens et al., 2015; DeSmet et al., 2016; Schneider et al., 2012). However, unlike traditional bullying, cyberbullying can occur 24 h a day, at anytime and anywhere, enables the anonymity of the aggressor(s) and has a larger potential audience (Elipe et al., 2018; Kowalski et al., 2014).

Research on cyberbullying shows varying prevalence rates across countries. Recently, a United Nations International Children’s Emergency Fund report (UNICEF, 2019) involving more than 170.000 young people (13–24 years old) across 30 countries revealed that one in three experienced cyberbullying and that one in five children skipped school because of it.

The global COVID-19 pandemic posed additional challenges to the safety and well-being of young people who were forced to leave classrooms and engage in online learning. In 2020, the Pew Internet Survey revealed that 41% of Americans were harassed online (Vogels et al., 2021), and according to the Microsoft’s Digital Civility Index for 2021, 82% of youth and adults from 18 countries revealed that the lockdown caused by the pandemic deteriorated the online civility (Beauchere, 2021). This is in line with data collected from social media users during the pandemic, revealing a significant increase in abusive content during the lockdown restrictions (Babvey et al., 2021). Similarly, research analysing public tweets on Twitter from January 2020 to June 2020 revealed an increase in cyberbullying (Das et al., 2020). Thus, based on these findings, we expect high levels of prevalence of cyberbullying during the two major lockdowns that occurred in Portugal (March to May 2020 and January to April 2021). Indeed, previous research conducted with young people (9–17 years old) in this national context before the COVID-19 pandemic showed (Ponte & Batista, 2019) higher rates of cyberbullying, compared to face-to-face bullying. The prevalence of cyberbullying victimization was 24%, and 16% of cyberbullying perpetration (Smahel et al., 2020). Moreover, the most reported aggression was receiving hurtful digital messages (64%) and almost three-quarters of young people revealed feeling uncomfortable as a result of cyberbullying experience (Ponte & Batista, 2019; Smahel et al., 2020). Based on previous research showing the detrimental impact of cyberbullying on mental health, we expected high levels of symptoms of psychological distress for those who experienced cyberbullying.

## Predictors of cyberbullying perpetration and victimization

Research focusing on cyberbullying shows that there are several factors commonly associated with cyberbullying perpetration and victimization (e.g., gender, socioeconomic status, age, minority identity, etc.; Kowalski et al., 2014). Some studies have found gender differences in terms of cyberbullying perpetration and victimization (e.g., Kowalski & Limber, 2007), but the results are not consistent (Saleem et al., 2022). Whereas some studies show that girls are more likely to experience cyberbullying, compared to boys (Li, 2007), others found small or no gender differences in terms of victimization (Hinduja & Patchin, 2008; Li, 2006; Smith et al., 2008), suggesting that these differences are less clear for this form of bullying (Smith et al., 2018). Research also shows that boys are more likely to perpetrate cyberbullying, compared to girls (Piccoli et al., 2020). A meta-analysis conducted with 25 studies revealed that males were associated with higher levels of cyberbullying perpetration, whereas being a female was significantly associated with being more likely to experience cyberbullying (Guo, 2016). This is also consistent with research conducted in Portugal revealing higher rates of experience of cyberbullying among females and higher rates of cyberbullying perpetration among males (Carvalho et al., 2021). Thus, in the current research, we will examine the relation between sex and involvement in cyberbullying incidents.

Cyberbullying has also been related to age. Some studies show that older youth (i.e., 15–17 years) were more likely to be involved in cyberbullying perpetration than younger youth (i.e., 10–14 years), but no differences were found in experiencing cyberbullying (Guo, 2016; Ybarra & Mitchell, 2004a). Researchers argue that younger individuals tend to solve bullying by fighting, while older ones tend to extend what happens offline to online bullying (Chen et al., 2017; Perren et al., 2010). Besides younger youth, research shows that cyberbullying also occurs quite frequently among college students, with more than 30% indicating that their first cyberbullying experience was in college (Kowalski et al., 2012). Importantly 43% of those who had been cyberbullied in middle school, high school, and college, revealed that most of the cyberbullying was experienced during college (Kowalski et al., 2012). There is also evidence of high levels of cyberbullying experience among college students in the Portuguese context. For instance, one study conducted with 349 university students revealed that 28% experienced cyberbullying (Francisco et al., 2015). Thus, in this research, we examine the relationship between participants’ education level and involvement in cyberbullying incidents.

Experiences of cyberbullying are also very prevalent among youth with certain characteristics and group-based minority identities (e.g., obese youth, ethnic minority youth, and sexual minority youth; Earnshaw et al., 2018). Recent data revealed increased discrimination practices and hate speech during the COVID-19 pandemic against certain minority groups (e.g., LGBTQ people, national or ethnic minorities, Roma people, and migrants; Marsal et al., 2020; United Nations High Commission for Human Rights, 2020), leading to increased insecurity, social exclusion, isolation, and stigmatization. In Portugal, a recent study developed with 14–19 years old youth in 2020 and 2021 revealed that LGBTQ + youth more frequently experienced forms of aggression (e.g., bullying and cyberbullying), compared to heterosexual and cisgender youth (Fernandes et al., 2022). Considering that bullying is particularly prevalent in socially marginalized groups, we will also explore if students who identify as members of minority groups (e.g., sexual minorities) report higher levels of cyberbullying victimization (both prevalence and psychological distress).

Social economic status (SES) has also been associated with both the perpetration and victimization of bullying, albeit in opposite ways. Research shows higher SES is associated with higher levels of cyberbullying perpetration (e.g., Kowalski et al., 2014; Ybarra & Mitchell, 2004b). In contrast, meta-analytical evidence shows that having lower levels of SES is associated with higher rates of bullying victimization (Tippett & Wolke, 2014). Similarly, research reveals that adolescents of lower SES families report a higher likelihood of experiencing cyberbullying (e.g., Hong et al., 2016). Less is known about SES and bullying perpetration and victimization in the Portuguese context, although one study showed family lower SES was associated with higher rates of both bullying victimization and perpetration among adolescents (Gaspar et al., 2014; Pereira et al., 2004). In the current research, we will examine the relation between SES and involvement in cyberbullying incidents (as perpetrators and victims) during the two lockdown periods.

Finally, an additional predictor of cyberbullying involvement is previous experience with face-to-face, traditional bullying (Kowalski et al., 2012). Research conducted on the overlap between involvement in both types of bullying (e.g., Hinduja & Patchin, 2008; Kowalski et al., 2014; Perren et al., 2010; Raskauskas & Stoltz, 2007; Smith et al., 2008), shows that perpetrators and those who experienced cyberbullying also experienced and were perpetrators of traditional bullying (Smith et al., 2008). In line with this, research shows that cyberbullying perpetrators may use social media to publicly humiliate their traditional bullying aggressor (Kowalski et al., 2014). In the Portuguese context, there is also evidence adolescents’ involvement in bullying is associated with involvement in cyberbullying episodes (Carvalho et al., 2021). Building on this research, we explore if previous involvement in traditional bullying is associated with involvement in cyberbullying incidents (Study 2).

## Buffering the effects of cyberbullying: the role of social and parental support

Recent approaches consider bullying as a complex behavior, that involves an ecological context, highlighting the role of different social and group factors in reducing the risk of involvement in bullying or in mitigating its negative effects on youth (e.g., Hong et al., 2020; Zhao et al., 2021). One protective factor is the social support that may derive from different sources (e.g., peers, friends, teachers, parents) and work as a buffering factor against bullying negative outcomes (Hellfeldt et al., 2020). For instance, high levels of social (i.e., from peers and teachers) and parental support among those who had experienced bullying have been shown to positively influence youth well-being and to reduce internalizing problems and substance use (e.g., Flaspohler et al., 2009; Hong et al., 2020). Similarly, parental support (e.g., higher levels of parental communication) also buffers adolescents against the negative effects of bullying (Ledwell & King, 2013).

Parental and social support can also be especially protective for minority youth (e.g., António & Moleiro, 2015; Espelage et al., 2008; Hong & Garbarino, 2012). For instance, research conducted in the Portuguese context shows that social and parental support moderated the effects of victimization on psychological distress, including suicidal ideation and school difficulties among youth experiencing homophobic bullying (António & Moleiro, 2015).

The protective role of social support has also been reported in the context of cyberbullying (e.g., Hellfeldt et al., 2020; Machmutow et al., 2012). Specifically, studies revealed that higher levels of social and parental support are related to higher levels of well-being among those who experienced cyberbullying and cyberbullying bully-victims (i.e., those who are both a perpetrator and experienced cyberbullying, Hellfeldt et al., 2020).

Building on this research, we examine whether social and parental support buffer the negative relation between cyberbullying victimization and youth well-being (i.e., psychological distress). Specifically, we explore if those who experienced cyberbullying but have higher levels of social and parental support show lower symptoms of psychological distress (e.g., anxiety) than those with lower levels of social and parental support.

## Study 1

This correlational study examined cyberbullying prevalence and its predictors, symptoms of psychological distress, as well as the buffering role of social support. We focused specifically on the Portuguese context during the first lockdown declared by the government on March 2020. Overall, based on previous research, we expected that those who experienced cyberbullying would report higher levels of psychological distress, compared to those who did not experience cyberbullying (H1); and that those who experienced cyberbullying with higher levels of social support would show lower levels of psychological distress (e.g., anxiety; H2). Moreover, we expected that students belonging to minority groups (e.g., sexual minorities) would report higher levels of cyberbullying victimization (H3).

## Method

### Participants and procedure

Four hundred and eighty-five students from Portugal (83.7% females), aged between 16 and 34 (*M =* 18.4, *SD =* 2.36), participated in this study. Approximately 1.4% of the students were in middle school (7th to 9th years); 62.5% were in high school (10th to 12th years); and 36.1% were in college. Three hundred and fifty-four participants identified as heterosexual, 62 as bisexual, 27 as gay or lesbian, and the remaining did not answer or had doubts as to their sexual orientation. Regarding participants’ household income during the pandemic, 15.3% revealed having a low income and 84.7% considered their income allowed them to live comfortably.

The survey was approved by the institutional Ethics Committee and conducted in accordance with the ethical standards of the American Psychological Association, the Declaration of Helsinki, and the European General Data Protection Regulation. All students who participated in the study had to provide previous informed consent and before participating they were informed that their participation was voluntary and anonymous. The survey was conducted online (June 2020 - July 2020) and an invitation to participate in the study was sent to students’ associations and was also shared through social media channels. After completing the survey, participants were debriefed and thanked for their participation.

### Measures

Participants indicated, at the beginning of the survey, their age, sex, sexual orientation, SES, and level of education^1^, and were provided with a short definition of bullying and cyberbullying^2^ after the demographics.

#### Cyberbullying: victim, bully, bystander

Participants indicated, on a 3-point scale (1 = never, 2 = sometimes and 3 = often), the frequency of their involvement in cyberbullying behaviors as victims, bully, and bystander (College Cyberbullying Questionnaire; Francisco, 2012; Martins et al., 2014). They were presented with 10 statements describing diverse aggressive behaviors or actions related to each subscale: victim, bully, and bystander (e.g., Victim subscale: “Harassed me with sexual content”). Following Francisco (2012), item 10 (“other”) was removed from all subscales, since it is one of the items that contribute less to the internal consistency of the subscales. The final subscales, involving 9 items each, were aggregated in 3 indexes, (victim, α = 0.85; bully, α = 0.79; bystander, α = 0.92, where higher values represent the higher frequency of these behaviors). Following previous research (Martins et al., 2014; Vivolo-Kantor et al., 2014) we then created a binary score ranging from 0 (never was involved in cyberbullying behaviors) vs. 1 (was sometimes/often involved in cyberbullying behaviors). Thus, a score of zero denotes no frequency of bullying versus a score of 1 denotes the frequency of bullying as sometimes/often.

#### Cyberbullying: emotions and motivations

Participants indicated, in a multiple-answer question, whether they felt 16 emotions arising from their involvement in cyberbullying (e.g., joy, indifference, College Cyberbullying Questionnaire, Francisco, 2012; Martins et al., 2014), and the reasons that led them to cyber-attack (6 options, e.g., revenge of previous episodes; so that the group would accept me).

#### Symptoms of psychological distress

A reduced version of the CORE-OM for adolescents was used (Sales et al., 2012) to assess global psychological well-being. Participants were asked to indicate how often they experienced the symptoms described in 10 items, on a 5-point scale (1 = never; 5 = very frequently; e.g., “I have felt tense, anxious or nervous”; “I have thought of hurting myself” α = 0.83). We computed a mean score with higher values indicating greater levels of psychological distress.

#### Social support

We used two items from the Kidscreen Quality of Life European survey adapted to the Portuguese population by Gaspar and Matos (2008). Participants indicated, on a 5-point scale (1 = never, 5 = very frequently), the extent to which they felt that their friends supported them (2 items; e.g., “You felt you could trust your friends”; *r* = .72). We computed a composite score of social support with higher values indicating higher levels of social support.

#### Parental support

We used a 2-item measure to assess parental support levels (Espelage et al., 2008). Participants indicated, on a 5-point scale (1 = never, 5 = very frequently), to what extent they felt that their parents worried about them and were available when needed (e.g., “You feel like your parents care about you.”; *r* = .74). We computed a composite score of parental support, where higher values indicate higher levels of parental support.

## Results and discussion

### Characteristics and prevalence of cyberbullying

Most participants (61%) reported experiencing cyberbullying in the last 3 months and 40.8% reported being perpetrators. Most students surveyed (86%) reported they had witnessed someone else being cyberbullied, although only half of them (51%) did something to stop the incident. The most frequent behaviors experienced in the role of victim were being mocked, being insulted and being victim of rumors. Similarly, the behaviors most frequently practiced in the role of perpetrator were also mockery and insult (see supporting information). The emotions most frequently reported by those who experienced cyberbullying were insecurity, anger, and sadness and by the perpetrators were indifference, guilt, and anger. As for the motives identified by the perpetrators, the most indicated reason was “for fun” (48.8%), followed by the “revenge of previous episodes” (28.7%).

### Predictors of cyberbullying perpetration and victimization: sex, education level, sexual orientation and SES

We conducted 4 Brown-Forsythe Tests^3^ to explore differences in participants’ scores of cyberbullying victimization, perpetration, and observation according to sex, education level, sexual orientation and SES.

Regarding participants’ sex and education level (middle school vs. high school vs. college), no significant results were found on cyberbullying victimization, perpetration and observation (see Table 1).

**Table 1:** Means and standard deviations of cyberbullying victimization, perpetration and observation by sex, education level, sexual orientation, and socio-economic status

Sex	Female			Male				
	*M*		*SD*	*M*		*SD*	*BF*	*p* value
Victimization	1.36		0.29	1.49		0.42	3.95	0.05
Perpetration	1.19		0.14	1.29		0.34	3.33	0.08
Observation	1.86		0.48	1.87		0.52	0.02	0.90
Education level	Middle School		High School		College			
	*M*	*SD*	*M*	*SD*	*M*	*SD*	*BF*	*p* value
Victimization	1.62	0.23	1.37	0.31	1.39	0.34	1.47	0.25
Perpetration	1.80	1.04	1.21	0.17	1.19	0.13	0.98	0.50
Observation	2.16	0.69	1.86	0.47	1.86	0.48	0.72	0.51
Sexual orientation	Heterosexual			LGB+				
	*M*		*SD*	*M*		*SD*	*BF*	*p* value
Victimization	1.35		0.29	1.46		0.34	6.16	0.01*
Perpetration	1.21		0.17	1.21		0.15	0.08	0.78
Observation	1.82		0.48	1.99		0.46	10.29	0.00**
SES	Low socio-economic status			High socio-economic status				
	*M*		*SD*	*M*		*SD*	*BF*	*p* value
Victimization	1..42		0.37	1.36		0.30	1.43	0.24
Perpetration	1.28		0.22	1.20		0.21	3.85	0.06
Observation	1.98		0.51	1.85		0.48	3.66	0.06

* p < .05 ** *p* < .001

Participants’ sexual orientation revealed a significant main effect on cyberbullying victimization, *BF*(1, 118) = 6.158, *p* = .014, η^2^ = 0.026, and observation, *BF*(1, 183) = 10.285, *p* = .002, η^2^ = 0.024 (see Table 1). Pairwise comparisons revealed that, as hypothesized, LGB + students reported higher levels of victimization and observation of cyberbullying, compared to heterosexual students. No significant results were found with regard to cyberbullying perpetration.

Regarding participants’ socio-economic status, no significant results were found on cyberbullying victimization, perpetration and observation (see Table 1).

### Symptoms of psychological distress

We conducted a Brown-Forsythe Test to compare symptoms of psychological distress among those who experienced and those who did not experience cyberbullying. Supporting our hypothesis, the results showed statistically significant differences between the two groups in 9 of the 10 symptoms of psychological distress measured in the questionnaire (see Table 2). Overall, those who experienced cyberbullying, when compared with those who did not experience cyberbullying, reported greater average levels of symptoms of psychological distress (e.g., “you thought of hurting yourself”; “you felt angry or nervous”).

**Table 2:** Mean differences on symptoms of psychological distress for those who experienced and who did not experience cyberbullying

		Experienced		Did not experience	
Symptoms of psychological distress	*BF*	*M*	*SD*	*M*	*SD*
You felt angry or nervous	27.20**	3.70	0.93	3.24	0.96
You didn’t feel like talking to anyone	20.21**	3.31	1.18	2.84	1.11
You felt you were able to deal with things that went wrong	5.08*	2.84	0.98	2.63	0.99
You thought of hurting yourself	26.42**	1.73	1.12	1.30	0.74
You felt the courage to ask someone for help	1.13	3.39	1.25	3.27	1.31
Your thoughts and feelings made you feel bad or suffer	43.55**	3.46	1.25	2.70	1.23
You felt your problems were too much for you	59.56**	3.30	1.31	2.39	1.26
You had difficulty falling asleep or staying asleep (all night)	26.54**	3.59	1.31	2.95	1.39
You felt sad	47.13**	3.69	1.05	3.03	1.04
You did all the things you wanted	2.83	3.36	0.99	3.19	1.07

*p < .05 **p < .001

### The moderator role of social support and parental support

We used PROCESS bootstrapping macro to explore if social and parental support moderated the relation of cyberbullying victimization and symptoms of psychological distress and suicidal ideation (Model 1; Hayes, 2013). Cyberbullying victimization was entered as the predictor, symptoms of psychological distress, and suicide ideation as separate outcomes, and social and parental support were entered as separate moderators. Four models were tested, one per outcome and moderator.

#### Parental support

**Fig. 1:**
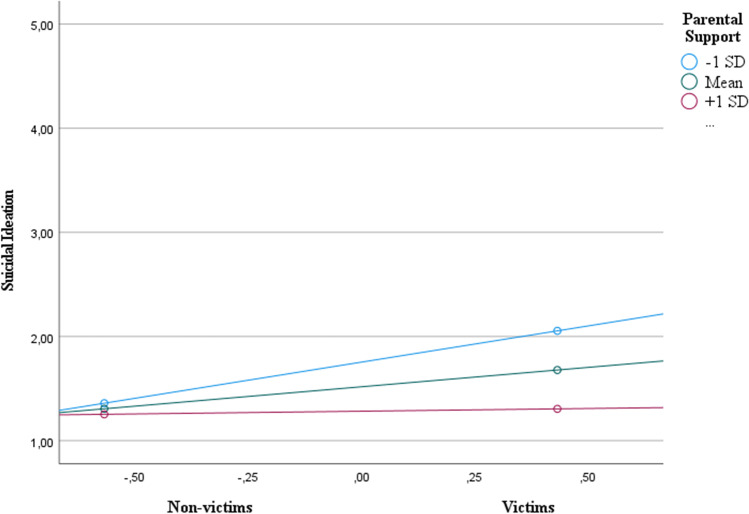
Moderator effect of parental support on the effect of cyberbullying victimization on the suicidal ideation (Study 1)

Results revealed that cyberbullying victimization was positively related to suicide ideation; *b* = 0.37, *p* < .001, that is, the more students reported experiencing cyberbullying, the more they thought of hurting themselves (see Table 3). The direct relation of parental support with suicidal ideation (*b* = -0.22, *p* < .001) was also reliable, suggesting that the more parental support they received, the fewer students thought of hurting themselves. As predicted, there was a significant interaction between parental support and cyberbullying victimization, *b* = − 0.30, *p* < .001 (H2; see Fig. 1). Cyberbullying victimization was positively related to suicide ideation only for those with low parental support (-1 SD; *b* = 0.70, 95% CI [0.44, 0.95]), and with an average level of parental support (*b* = 0.37, 95% CI [0.20, 0.54]), but not for participants with higher levels of parental support (+ 1 SD; *b* = 0.05, 95% CI [-0.18, 0.29]). Regarding psychological distress, no significant moderation effects were found.

**Table 3:** Moderator effect of parental support on the effect of victimization on symptoms of psychological distress (Study 1)

	Y (symptoms of psychological distress)			Y (suicide ideation)				
	Coeff.	*SE*	*p*	Coeff.	*SE*		*p*	
Constant	3.02**	0.03	0.00	1.52**	0.04		0.00	
(X) Cyberbullying victimization	0.40**	0.06	0.00	0.37**	0.09		0.00	
W (Parental support)	-0.23**	0.03	0.00	-0.22**	0.04		0.00	
X x W	-0.10	0.06	0.10	-0.30*	0.08		0.02	
	*R*^2^ = 0.22 * F* (3, 473) = 44.55, *p* < .001			*R*^2^ = 0.14 * F* (3, 472) = 25.03, *p* < .001				

The values are unstandardized regression coefficients

* p < .05 ** *p* < .001

#### Social support

The models for social support did not reveal significant effects for symptoms of psychological distress and suicide ideation (see Table 4).

**Table 4:** Moderator effect of social support on the effect of victimization on symptoms of psychological distress (Study 1)

	Y (symptoms of psychological distress)			Y (suicide ideation)				
	Coeff.	*SE*	*p*	Coeff.		*SE*		*p*
Constant	3.02**	0.03	0.00	1.54**	0.04		0.00	
(X) Cyberbullying victimization	0.43**	0.06	0.00	0.38**	0.09		0.00	
W (Social support)	-0.23**	0.03	0.00	-0.25**	0.04		0.00	
X x W	-0.07	0.07	0.28	-0.12	0.09		0.18	
	*R*^2^ = 0.20 * F* (3, 473) = 0.75, *p* < .001			*R*^2^ = 0.11 * F* (3, 472) = 20.43, *p* < .001				

Note The values are unstandardized regression coefficients

* p < .05 ** *p* < .001

## Discussion

Overall, the results of Study 1 supported our hypotheses and are in line with previous research, showing that LGB + students reported higher levels of victimization, compared to heterosexual students (e.g., Llorent et al., 2016). Importantly, as predicted, symptoms of psychological distress during lockdowns (e.g., sadness and loneliness) were higher for those who experienced than for those who did not experience cyberbullying. Also, parental support moderated the effects of cyberbullying victimization on suicide ideation. Thus, the level of suicide ideation by those who experienced cyberbullying was greater when parental support was low. These results are further discussed in the General Discussion.

## Study 2

The main goal of Study 2 was to replicate Study 1, aiming to better understand the impact of cyberbullying on youth with a more diverse sample of Portuguese students. Specifically, we conducted a correlational study to examine again the prevalence, predictors, and symptoms of psychological distress associated with the experience of cyberbullying, this time during the second lockdown period (January - April 2021). Similar to Study 1, we expected that those who experienced cyberbullying would report higher levels of psychological distress, compared to those who did not experience cyberbullying (H1); and that those who experienced cyberbullying with higher levels of social support would show lower levels of psychological distress (e.g., anxiety) than those with lower levels of social and parental support (H2). Moreover, we expected that students from minority groups (e.g., sexual minorities) would report higher levels of cyberbullying victimization (H3). Finally, in this study we included a measure of previous involvement in traditional bullying to understand whether an association exists between previous involvement in traditional bullying and involvement in cyberbullying incidents. Based on previous research, we expected that previous involvement in traditional bullying will be associated with more involvement in cyberbullying incidents (H4).

## Method

### Participants and procedure

Nine hundred and fifty-two students from Portugal (67.7% females), aged between 13 and 30 (*M =* 19.4, *SD =* 3.51), participated in this study. Approximately 11.3% were in middle school (7th to 9th years); 34.2% were in high school (10th to 12th years); and 54.5% were in college. Seven hundred and sixteen participants identified as heterosexual, 130 as bisexual or pansexual, 30 as gay or lesbian and the remaining did not answer or had doubts as to their sexual orientation. Regarding participants’ household income during the pandemic, 19.1% revealed having a low income and 80.9% considered their income allowed them to live comfortably.

The survey was approved by the institutional Ethics Committee and conducted in accordance with the ethical standards of the American Psychological Association, the Declaration of Helsinki, and the European General Data Protection Regulation. All students who participated in the study had to provide previous informed consent and before participating they were informed that their participation was voluntary and anonymous. The survey was developed online and an invitation to participate in the study was sent to students’ associations and was also shared through social media. After completing the survey, participants were debriefed and were informed they could participate in a lottery to win a 25€ voucher as a way of thanking their participation.

### Measures

Cyberbullying, social, and parental support, and symptoms of psychological distress were assessed with the same measures used in Study 1.

#### Past traditional bullying involvement

We adapted 4 items from previous research (e.g., Raskauskas, 2010). Participants indicated, on a 5-point scale (1 = never, 2 = only once or twice, 3 = occasionally, 4 = about once a month, and 5 = once a week or more), to what extent they had experienced four types of face-to-face, traditional bullying (i.e., physical, verbal, exclusion, and gossip) in the last 2 school years (α = 0.77).

## Results and discussion

### Characteristics and prevalence of cyberbullying

Most participants (71%) reported having experienced cyberbullying in the last 4 months and 39% reported being perpetrators. Most students (80%) reported they had witnessed someone else being cyberbullied, although only 60% did something to stop the incident.

The most frequent behaviors experienced were being mocked and being insulted and the behaviors most frequently practiced were also mockery and insult (see supporting information). The emotions more frequently reported by those who experienced cyberbullying were insecurity, sadness and worry, and by the perpetrators were indifference, guilt, and superiority. As for the motives identified by the perpetrators, the most indicated reasons were “for fun” (52%) and “revenge of previous episodes” (31%).

### Predictors of cyberbullying perpetration and victimization: sex, education level, sexual orientation, SES, and previous involvement in traditional bullying

We conducted Brown-Forsythe Tests^4^ to explore differences between female and male students in terms of their scores on cyberbullying victimization, perpetration, and observation (see Table 5). Results revealed a significant effect of participants’ sex on cyberbullying victimization, *BF*(1, 502) = 14.539, *p* < .001, η^2^ = 0.018, perpetration, *BF*(1, 241) = 14.489, *p* < .001, η^2^ = 0.043, and observation, *BF*(1, 352) = 13.423, *p* < .001, η^2^ = 0.018. Pairwise comparisons revealed that female participants reported higher levels of victimization and observation, than male participants. Additionally, male participants reported higher levels of perpetration, than female participants.

**Table 5:** Means and standard deviations of cyberbullying victimization, perpetration and observation by sex, education level, sexual orientation, and socio-economic status

Sex	Female			Male				
	*M*		*SD*	*M*		*SD*	*BF*	*p *value
Victimization	1.63		0.44	1.51		0.35	14.54	0.00**
Perpetration	1.23		0.15	1.30		0.22	14.49	0.00**
Observation	1.92		0.51	1.77		0.52	13.42	0.00**
Education level	Middle School		High School		College			
	*M*	*SD*	*M*	*SD*	*M*	*SD*	*BF*	*p* value
Victimization	1.55^b^	0.40	1.68^a^	0.44	1.52^b^	0.37	10.55	0.00**
Perpetration	1.28^ab^	0.22	1.28^a^	0.19	1.23^b^	0.16	2.92	0.06
Observation	1.80^ab^	0.52	1.97^a^	0.53	1.86^b^	0.48	4.63	0.01*
Sexual orientation	Heterosexual			LGB+				
	*M*		*SD*	*M*		*SD*	*BF*	*p* value
Victimization	1.54		0.40	1.74		0.43	26.29	0.00**
Perpetration	1.26		0.19	1.25		0.15	0.33	0.86
Observation	1.83		0.50	2.02		0.49	19.35	0.00**
SES	Low socio-economic status			High socio-economic status				
	*M*		*SD*	*M*		*SD*	*BF*	*p* value
Victimization	1.72		0.42	1.56		0.40	17.10	0.00**
Perpetration	1.24		0.19	1.26		0.18	0.83	0.37
Observation	1.97		0.50	1.86		0.51	6.01	0.02*

Means with different subscripts in each column indicate differences at *p* < .050

* p < .05 ** *p* < .001

Regarding differences in cyberbullying victimization, perpetration, and observation between participants’ education level (middle school vs. high school vs. college; see Table 5), results revealed a significant effect on cyberbullying victimization, *BF*(2, 325) = 10.548, *p* < .001, η^2^ = 0.034, and observation *BF*(2, 254) = 4.634, *p* = .01, η^2^ = 0.014. Pairwise comparisons revealed that high school students reported higher levels of victimization, compared to middle school and college students. High school students also reported higher levels of observation, compared to college students.

Participants’ sexual orientation revealed a significant effect on cyberbullying victimization, *BF*(1, 245) = 26.292, *p* < .001, η^2^ = 0.042, and observation, *BF*(1, 326) = 19.349, *p* < .001, η^2^ = 0.025 (see Table 5). Pairwise comparisons revealed that, as hypothesized, LGB + students reported higher levels of victimization and observation of cyberbullying, compared to heterosexual students. No significant results were found with regard to cyberbullying perpetration.

Regarding participants’ SES, results revealed a significant effect on cyberbullying victimization, *BF*(1,229) = 17.103, *p* < .001, η^2^ = 0.027, and observation *BF*(1, 223) = 6.007, *p* = .015, η^2^ = 0.08 (see Table 5). Pairwise comparisons revealed that students with high SES reported lower levels of cyberbullying victimization, compared to students with low SES. Also, students with high SES reported lower levels of cyberbullying observation, compared to students with low SES. No significant results were found with regard to cyberbullying perpetration.

Lastly, regarding previous involvement in traditional bullying, as hypothesized, results revealed a significant effect on victimization, (R^2^ = 0.21, *F*(1, 639) = 172.98, *p* < .000). Specifically, it was found that previous involvement in traditional bullying significantly predicted cyberbullying victimization (β = 0.227, *p* < .000). No significant results were found with regards to cyberbullying perpetration (β = 0.016, *p* = .121).

### Symptoms of psychological distress

We conducted Brown-Forsythe Test to compare the average of symptoms of psychological distress among those who experienced and those who did not experience cyberbullying. Supporting our hypothesis, results revealed statistically significant differences between those who experienced and those who did not experience cyberbullying in 9 of the 10 symptoms of psychological distress measured in the questionnaire (see Table 6). Those who experienced cyberbullying, compared with those who did not experience it, reported higher symptoms of psychological distress (e.g., “you thought of hurting yourself”; “you felt angry or nervous”).

**Table 6:** Mean differences on symptoms of psychological distress for those who experienced and who did not experience cyberbullying

		Experienced		Did not experience	
Symptoms of Psychological Distress	*BF*	*M*	*SD*	*M*	*SD*
You felt angry or nervous	25.79**	3.54	0.99	3.14	1.10
You didn’t feel like talking to anyone	35.97**	3.28	1.14	2.79	1.13
You felt you were able to deal with things that went wrong	3.88*	2.86	1.03	2.72	0.94
You thought of hurting yourself	99.41**	2.01	1.28	1.32	0.79
You felt the courage to ask someone for help	3.98*	3.43	1.21	3.24	1.33
Your thoughts and feelings made you feel bad or suffer	60.34**	3.43	1.22	2.70	1.32
You felt your problems were too much for you	48.31**	3.25	1.32	2.58	1.33
You had difficulty falling asleep or staying asleep (all night)	54.97**	3.37	1.38	2.63	1.38
You felt sad	49.93**	3.57	1.08	2.99	1.14
You did all the things you wanted	1.39	3.38	1.01	3.29	1.04

*p < .05 **p < .001

### The moderator role of social support and parental support

We used PROCESS bootstrapping macro to explore if social and parental support moderated the relation of cyberbullying victimization and symptoms of psychological distress, and suicidal ideation (Model 1; Hayes, 2013). Cyberbullying victimization was entered as the predictor, symptoms of psychological distress, and suicide ideation as separate outcomes, and social and parental support were entered as separate moderators. Four models were tested, one per outcome and moderator.

#### Parental support

**Fig. 2:**
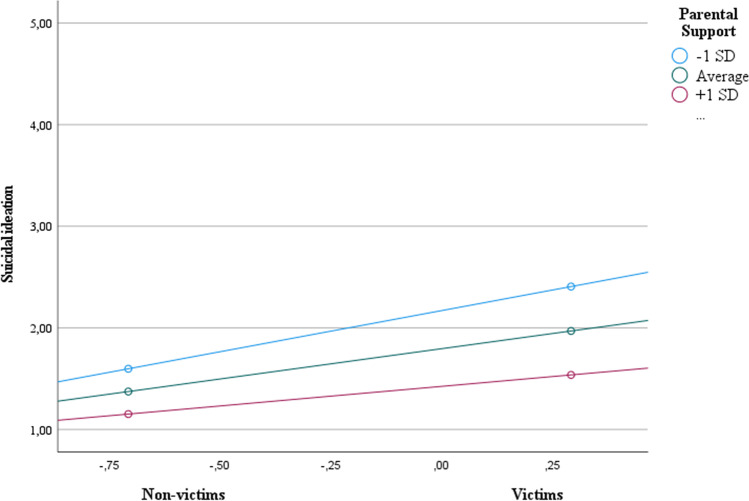
Moderator effect of parental support on the effect of cyberbullying victimization on the suicidal ideation (Study 1)

Results revealed that cyberbullying victimization was positively related to suicide ideation, *b* = 0.60, *p* < .001, that is, the more students experienced cyberbullying, the more they thought of hurting themselves (see Table 7). The direct relation of parental support with suicidal ideation (*b* = -0.33, *p* < .001) was also reliable, suggesting that the more parental support they received, the fewer students thought of hurting themselves. As predicted, there was a significant interaction between parental support and cyberbullying victimization, *b* = − 0.19, *p* = .02 (H2; see Fig. 2). Cyberbullying victimization was positively related to suicide ideation but stronger for those with low parental support (-1 SD; *b* = 0.81, 95% CI [0.55, 1.07]), and lower for those with an average level of parental support (*b* = 0.60, 95% CI [0.44, 0.76]), and with higher levels of parental support (+ 1 SD; *b* = 0.39, 95% CI [0.17, 0.60]). Regarding psychological distress, no significant moderation effects were found (see Table 7).

**Table 7:** Moderator effect of parental support on the effect of victimization on symptoms of psychological distress (Study 2)

	Y (symptoms of psychological distress)			Y (suidice ideation)				
	Coeff.	*SE*	*p*	Coeff.		*SE*		*p*
Constant	3.06**	0.02	0.00	1.80**	0.04		0.00	
(X) Cyberbullying victimization	0.33**	0.05	0.00	0.60**	0.08		0.00	
W (Parental support)	-0.27**	0.02	0.00	-0.33**	0.03		0.00	
X x W	0.04	0.05	0.39	-0.19*	0.08		0.02	
	*R*^2^ = 0.22 * F* (3, 899) = 85.61, *p* < .001			*R*^2^ = 0.18 * F* (3, 898) = 64.81, *p* < .001				

*Note.* The values are unstandardized regression coefficients

* p < .05 ** *p* < .001

#### Social support

**Fig. 3:**
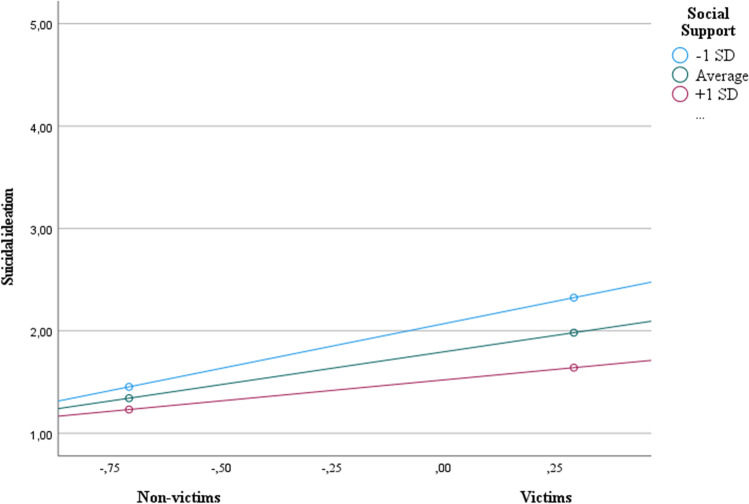
Moderator effect of social support on the effect of cyberbullying victimization on suicidal ideation (Study 2)

Results revealed that cyberbullying victimization was positively related to suicide ideation, *b* = 0.64, *p* < .001, that is, the more students experienced cyberbullying, the more they thought of hurting themselves (see Table 8). The direct relation of social support with suicidal ideation (*b* = -0.26, *p* = .001) was also reliable, suggesting that the more social support they receive, the fewer students thought of hurting themselves. As predicted, there was a significant interaction between social support and cyberbullying victimization, *b* = − 0.22, *p* = .01 (H2; see Fig. 3). Cyberbullying victimization was positively related to suicide ideation but stronger for those with low social support (-1 SD; *b* = 0.87, 95% CI [0.61, 1.13]), and lower for those with an average level of social support (*b* = 0.64, 95% CI [0.48, 0.80]), and with higher levels of social support (+ 1 SD; *b* = 0.41, 95% CI [0.19, 0.63]). Regarding psychological distress, no significant moderation effects were found (see Table 8).

**Table 8:** Moderator effect of social support on the effect of victimization on symptoms of psychological distress (Study 2)

	Y (symptoms of psychological distress)			Y (suicide ideation)				
	Coeff.	*SE*	*p*	Coeff.		*SE*		*p*
Constant	3.06**	0.02	0.00	1.80**	0.04		0.00	
(X) Cyberbullying victimization	0.36**	0.05	0.00	0.64**	0.08		0.00	
W (Social support)	-0.26**	0.02	0.00	-0.26**	0.04		0.00	
X x W	-0.03	0.05	0.60	-0.22*	0.08		0.01	
	*R*^2^ = 0.19 * F* (3, 899) = 71.60, *p* < .001			*R*^2^ = 0.13 * F* (3, 898) = 45.90, *p* < .001				

The values are unstandardized regression coefficients

* p < .05 ** *p* < .001

In sum, these results supported our hypotheses and are consistent with previous work, showing that LGB + students reported higher levels of victimization, compared to heterosexual students (e.g., DeSmet et al., 2021), and male students reported higher levels of cyberbullying perpetration, compared to female students (e.g., Guo, 2016). Importantly, symptoms of psychological distress (e.g., sadness and loneliness) were higher for those who experienced cyberbullying than for those who did not experience it. Also, as predicted, parental and social support moderated the effects of cyberbullying victimization on suicide ideation. These results are further discussed in the General Discussion.

## General discussion

Two studies examined the prevalence of cyberbullying during two lockdowns in 2020/2021 stemming from the COVID-19 pandemic, focusing on its predictors, symptoms of psychological distress, and potential buffers of its negative impacts. Taken together, the results of the two studies provide strong evidence for the negative consequences of the two lockdowns for students, specifically the negative impact of cyberbullying on youth, and for the importance of parental and social support to buffer the negative effects of cyberbullying victimization on youth.

Consistent with previous research on the increase in abusive content during the lockdown restrictions (e.g., Babvey et al., 2021), our findings further illustrate a high prevalence of cyberbullying victimization and observation during lockdown periods resulting from the global COVID-19 pandemic. Specifically, results from both studies showed that over 60% of students experienced cyberbullying, suggesting that the COVID pandemic and the consequent switch to online learning platforms and social media use posed a risk for youth to be more exposed to cyberbullying. “Prank” and “revenge of previous episodes” were the most common motivations identified for cyberbullying perpetration. This is similar to findings from previous studies, showing that cyberbullying is commonly motivated by “fun”, with no apparent awareness of the seriousness and consequences of this type of behavior, and also by internal motivations (e.g., revenge), suggesting a continuity or transformation of bullying into cyberbullying (e.g., Gahagan et al., 2016; Martins et al., 2014).

Research focusing on cyberbullying shows that there are several factors commonly associated with cyberbullying perpetration and victimization (e.g., sex, and socioeconomic status; Kowalski et al., 2014). In this research, we examined the role of sex, education level, sexual orientation, socio-economic status, and previous involvement in traditional bullying as predictors of cyberbullying perpetration, victimization, and observation. In Study 1, no differences were found regarding cyberbullying victimization, perpetration and observation, and participants’ sex, while in Study 2, female participants reported higher levels of victimization, and male participants reported higher levels of cyberbullying perpetration. Indeed, some studies have found sex differences in terms of cyberbullying perpetration and victimization, with girls being more likely to experience cyberbullying, and boys being more likely to perpetrate cyberbullying (e.g., Guo, 2016; Kowalski & Limber, 2007; Li, 2006), while other studies found small or no sex differences (e.g., Smith et al., 2008). Future research could explore the differential impact of sex on cyberbullying incidents, which may also have implications for intervention, as all youth are highly attracted to information and communication technologies, with girls usually being more connected to social networking sites and boys to internet gaming (Smith et al., 2018).

Regarding participants’ education level, in Study 2 high school students were more frequently involved in cyberbullying incidents, compared to middle school and college students. This is consistent with previous research, showing that older youth, particularly those in high school, are more likely to be involved in cyberbullying, than younger youth and young adults (Chen et al., 2017; Martins et al., 2014). However, in Study 1, no significant results were found regarding participants’ education levels. One potential difference that may account for this result has to do with the imbalance number of participants per education level, with most students being in high school and in college, and very few in middle school, which we tried to overcome in Study 2.

Regarding sexual orientation as a predictor of involvement in cyberbullying incidents, supportive of our hypothesis our results showed that LGB + students more frequently experienced and observed cyberbullying incidents, compared to heterosexual students. This is in line with research showing that bullying is particularly prevalent in socially marginalized groups, such as sexual minorities (e.g., DeSmet et al., 2021; Llorent et al., 2016).

Considering students’ socioeconomic status, our findings revealed that students with low SES reported higher levels of involvement in cyberbullying incidents, compared to students with higher SES. Previous research shows that individuals with higher SES have more frequent access to technology and are associated with more cyberbullying behaviors (e.g., Kowalski et al., 2014; Wang et al., 2009), although recent research considers all youth over time have access to internet and mobile technology (e.g., Duarte et al., 2018). Our findings should be interpreted with caution, considering the specific pandemic period in which data was collected. During the two lockdown periods schools were closed and replaced by online learning platforms, thereby increasing internet usage for all students regardless of their socio-economic status. Thus, future studies could further explore the differential impact of socioeconomic status on cyberbullying involvement.

Consistent with prior research showing that previous experience with face-to-face traditional bullying predicts cyberbullying involvement (e.g., Kowalski et al., 2012), our findings further illustrate that those who experienced cyberbullying reported higher levels of traditional bullying victimization (i.e., physical, verbal, exclusion, and gossip). Indeed, this is in line with previous research, showing that an overlap exists between involvement on both types of bullying (e.g., Hinduja & Patchin, 2008), showing that young people have to deal with this problem not only within the school.

Importantly, in both studies, symptoms of psychological distress (e.g., suicidal ideation, sadness and loneliness) were higher for those who experienced than for those who did not experience, which is consistent with previous research showing the several negative effects of cyberbullying victimization on youth well-being (e.g., Flaspohler et al., 2009). Thus, it is important to focus on how to reduce the impact of cyberbullying and to explore protective factors that may mitigate cyberbullying negative effects on youth.

Overall, both studies showed evidence suggesting that social and parental support can reduce some of the negative effects of cyberbullying victimization. Specifically, in both studies parental support moderated the effects of cyberbullying victimization on suicide ideation. Consistent with previous research, the level of suicide ideation experienced by the victims of cyberbullying was greater when the parental support was low (e.g., António & Moleiro, 2015). In Study 2, social support also moderated the effects of cyberbullying victimization on suicide ideation. Results revealed that the detrimental effect of victimization on suicide ideation was greater when the victims had lower social support. These findings are consistent with previous work, showing that both social and parental support mitigate the impact of bullying victimization on youth (e.g., Flaspohler et al., 2009; Ledwell & King, 2013; Machmutow et al., 2012). However, in Study 1 social support did not moderate the effects of cyberbullying victimization on psychological distress. Research shows that social support may derive from different sources (e.g., peers, friends, teachers, parents; Hellfeldt et al., 2020), however, we only included social support from peers. Thus, future studies could explore the moderator role of social support from teachers as a buffer against the negative effects of cyberbullying. Future studies could also compare the relative efficacy of parental and social support, exploring if different underlying mechanisms account for their buffering effects against the negative impacts of cyberbullying victimization. Indeed, students with more social and parental support tend to more easily communicate and process negative social experiences (e.g., Ledwell & King, 2013). Thus, it is important to highlight the need to create social support networks for victims of cyberbullying, focusing, for instance, on those who witness these incidents (i.e., bystanders).

Indeed, research reveals that cyberbullying incidents are usually observed by other peers (e.g., Brody & Vangelisti, 2016). As in previous studies, our findings revealed a high prevalence of bystanders in cyberbullying episodes, although they indicated only intervening or reporting half of the episodes. Given the effectiveness of bystanders to stop bullying incidents and support the victims, it is important to create intervention programs aiming to promote helping behaviors among cyberbullying bystanders (Midgett et al., 2015).

Overall, our results showed consistent evidence regarding the negative impact of cyberbullying on youth, and also of the importance of parental and social support to buffer the negative effects of cyberbullying victimization on youth. The present research highlights the need to create social support networks for those who experience cyberbullying and to create more awareness programs to increase empathy and improve young people’s behaviors online.

## Limitations and future directions

Overall, our findings are consistent with previous empirical work and provide important insights into the influence of the lockdowns stemming from the COVID-19 pandemic on students’ aggressive behaviors online. However, the current studies had some limitations, which must be addressed. The correlational nature of our data did not allow us to test causal relations among the variables. To overcome this limitation, future studies could test these findings experimentally, or longitudinally. Additionally, in Study 1 the sample was composed mostly of female students, which we tried to overcome in Study 2 with a larger and more representative sample of Portuguese youth. Nevertheless, future research could replicate these findings using a more representative sample, exploring cyberbullying, for instance, among younger age groups and in a post-pandemic context. Furthermore, given the widespread use of information and communication technologies by all youth and young adults, it is important to develop prevention and intervention strategies specific to each target and consider their more frequent online behaviors (e.g., girls are usually more interested in social networking sites and boys in online gaming; Smith et al., 2018). Also, it is important to consider the cut-off point chosen for defining cyberbullying, which affected the calculation of victimization prevalence. Previous research has been adopting different cut-off points and response options and there is still no consensus on the best approach to measure bullying and cyberbullying (e.g., Cook et al., 2010; Saleem et al., 2022), which ultimately impacts the overall prevalence rates and the kind of information reported (Vivolo-Kantor et al., 2014). Indeed, one could argue that the category “Sometimes” could be too subjective, not reflecting clearly the repetitiveness involved in bullying. Thus, it is important to conduct future research aiming at replicating the current findings using different category response types for cyberbullying prevalence.

Despite these limitations, our findings contribute to the existing knowledge on online bullying, specifically during the pandemic context we lived in. With the shift to a new normal, where students are back to regular learning, but anticipating similar future threats, it is essential to create more awareness, and develop measures to protect children and youth from online violence, to reduce the sharing of violent messages and content online and to intervene with young people, especially on issues related to cyberbullying.

## Supplementary information

Below is the link to the electronic supplementary material.

ESM 1 (DOCX 18.2 KB)

## Data Availability

The datasets generated during and/or analysed during the current studies are not publicly available due to privacy or ethical restrictions but are available from the corresponding author on reasonable request.

## Footnotes

1 The questionnaire also included other measures that were not relevant for this study.

2 “To answer these questions, it is important to know that: Bullying is a word used to describe acts of physical (e.g., hitting, pushing, assaulting) or psychological (e.g., teasing, insulting, spreading rumors) violence, which are repeated over time, being practiced by one or more people with the aim of assaulting or intimidating another person. Cyberbullying consists of using technology, such as social networks, to harass, threaten or victimize another person repeatedly and intentionally.”

3 Due to the violation of the assumption of homogeneity of variance.

4 Due to the violation of the assumption of homogeneity of variance.
