# Supplementary material for: Cyberbullying during COVID-19 lockdowns: prevalence, predictors, and outcomes for youth
Source: Curr Psychol. 2023 Feb 18:1–17. Online ahead of print. doi: 10.1007/s12144-023-04394-7 (PMC9938688; doi:10.1007/s12144-023-04394-7)
Supplement: Supplementary file 1 — (DOCX 18.2 KB) [file 12144_2023_4394_MOESM1_ESM.docx]

**Supplementary material**

Cyberbullying during COVID-19 lockdowns’: Prevalence, predictors, and outcomes for youth

**Table 1**

*Experience of cyberbullying (Study 1)*

|  | Never  % | Sometimes  % | Often  % |
| --- | --- | --- | --- |
| Threatened me | 74.1 | 24.1 | 1.8 |
| Harassed me with sexual content | 53.7 | 41.2 | 5.1 |
| Rumors spread about my life | 51.8 | 41.2 | 6.9 |
| Impersonated me | 84.6 | 14.7 | 0.7 |
| Made fun of me | 41.6 | 48.9 | 9.5 |
| Insulted me | 43.1 | 47.8 | 9.1 |
| Showed that they had information about my life that could affect my psychological well-being | 69.7 | 27 | 3.3 |
| Revealed data about my private life | 81 | 16.8 | 2.2 |
| Used my image without permission | 79.9 | 17.9 | 2.2 |

**Table 2**

*Experience as perpetrator (Study 1)*

|  | Never  % | Sometimes  % | Often  % |
| --- | --- | --- | --- |
| Threatened | 82.8 | 16 | 1.2 |
| Harassed with sexual content | 96.9 | 2.5 | 0.6 |
| I spread rumors about the lives of others | 84.1 | 15.2 | 0.6 |
| I pretended to be someone else | 93.2 | 6.2 | 0.6 |
| I made fun of someone | 33.1 | 62.6 | 4.3 |
| Insulted | 40.7 | 56.2 | 3.1 |
| I have shown that I have information about another person that can affect their psychological well-being | 88.9 | 10.5 | 0.6 |
| I revealed data about someone else's private life | 93.2 | 6.2 | 0.6 |
| I used someone's image without permission | 88.7 | 10.7 | 0.6 |

**Table 3**

*Experience of cyberbullying (Study 2)*

|  | Never  % | Sometimes  % | Often  % |
| --- | --- | --- | --- |
| Threatened me | 56.6 | 39 | 4.4 |
| Harassed me with sexual content | 48 | 40.7 | 11.3 |
| Rumors spread about my life | 35.9 | 45.9 | 18.2 |
| Impersonated me | 80.3 | 17.7 | 2.1 |
| Made fun of me | 28.1 | 50.8 | 21 |
| Insulted me | 23.4 | 57.8 | 18.8 |
| Showed that they had information about my life that could affect my psychological well-being | 59.5 | 31.8 | 8.8 |
| Revealed data about my private life | 64.7 | 29.5 | 5.8 |
| Used my image without permission | 66.4 | 27.8 | 5.8 |

**Table 4**

*Experience as perpetrator (Study 2)*

|  | Never  % | Sometimes  % | Often  % |
| --- | --- | --- | --- |
| Threatened | 78.2 | 20.5 | 1.3 |
| Harassed with sexual content | 97.3 | 2.7 | 0 |
| I spread rumors about the lives of others | 79.1 | 20.7 | 0.3 |
| I pretended to be someone else | 85.6 | 14.4 | 0 |
| I made fun of someone | 31.6 | 64.9 | 3.5 |
| Insulted | 44.7 | 51.8 | 3.5 |
| I have shown that I have information about another person that can affect their psychological well-being | 91.1 | 8.4 | 0.5 |
| I revealed data about someone else's private life | 87.3 | 12.5 | 0.3 |
| I used someone's image without permission | 81.4 | 18.0 | 0.5 |
